# Characterization of the receptor-binding domain (RBD) of 2019 novel coronavirus: implication for development of RBD protein as a viral attachment inhibitor and vaccine

**DOI:** 10.1038/s41423-020-0400-4

**Published:** 2020-03-19

**Authors:** Wanbo Tai, Lei He, Xiujuan Zhang, Jing Pu, Denis Voronin, Shibo Jiang, Yusen Zhou, Lanying Du

**Affiliations:** 1 Lindsley F. Kimball Research Institute, New York Blood Center, New York, NY USA; 2 Beijing Institute of Microbiology and Epidemiology, Beijing, China; 3 Key Laboratory of Medical Molecular Virology (MOE/NHC/CAMS), School of Basic Medical Sciences, Fudan University, Shanghai, China

**Keywords:** 2019 novel coronavirus, SARS-CoV-2, spike protein, receptor-binding domain, viral inhibitor, cross-neutralization, Viral infection, Immunotherapy

## Abstract

The outbreak of Coronavirus Disease 2019 (COVID-19) has posed a serious threat to global public health, calling for the development of safe and effective prophylactics and therapeutics against infection of its causative agent, severe acute respiratory syndrome coronavirus 2 (SARS-CoV-2), also known as 2019 novel coronavirus (2019-nCoV). The CoV spike (S) protein plays the most important roles in viral attachment, fusion and entry, and serves as a target for development of antibodies, entry inhibitors and vaccines. Here, we identified the receptor-binding domain (RBD) in SARS-CoV-2 S protein and found that the RBD protein bound strongly to human and bat angiotensin-converting enzyme 2 (ACE2) receptors. SARS-CoV-2 RBD exhibited significantly higher binding affinity to ACE2 receptor than SARS-CoV RBD and could block the binding and, hence, attachment of SARS-CoV-2 RBD and SARS-CoV RBD to ACE2-expressing cells, thus inhibiting their infection to host cells. SARS-CoV RBD-specific antibodies could cross-react with SARS-CoV-2 RBD protein, and SARS-CoV RBD-induced antisera could cross-neutralize SARS-CoV-2, suggesting the potential to develop SARS-CoV RBD-based vaccines for prevention of SARS-CoV-2 and SARS-CoV infection.

## Introduction

Three highly pathogenic human coronaviruses (CoVs) have been identified so far, including Middle East respiratory syndrome coronavirus (MERS-CoV), severe acute respiratory syndrome (SARS) coronavirus (SARS-CoV), and a 2019 novel coronavirus (2019-nCoV), as previously termed by the World Health Organization (WHO).^1–3^ Among them, SARS-CoV was first reported in Guangdong, China in 2002.^4^ SARS-CoV caused human-to-human transmission and resulted in the 2003 outbreak with about 10% case fatality rate (CFR),^1^ while MERS-CoV was reported in Saudi Arabia in June 2012.^5^ Even though with its limited human-to-human transmission, MERS-CoV showed a CFR of about 34.4%.^2^ The 2019-nCoV was first reported in Wuhan, China in December 2019 from patients with pneumonia,^6^ and it has exceeded both SARS-CoV and MERS-CoV in its rate of transmission among humans.^7^ 2019-nCoV was renamed SARS-CoV-2 by Coronaviridae Study Group (*CSG)* of the *International Committee on Taxonomy of Viruses (ICTV)*,^8^ while it was renamed HCoV-19, as a common virus name, by a group of virologists in China.^9^ The disease and the virus causing it were named Coronavirus Disease 2019 (COVID-19) and the virus responsible for COVID-19 or the COVID-19 virus, respectively, by WHO.^3^ As of March 5, 2020, a total of 95,333 confirmed cases of COVID-19 were reported, including 3,282 deaths, in China and at least 85 other countries and/or territories.^7^ Currently, the intermediate host of SARS-CoV-2 is still unknown, and no effective prophylactics or therapeutics are available. This calls for the immediate development of vaccines and antiviral drugs for prevention and treatment of COVID-19.

A coronavirus contains four structural proteins, including spike (S), envelope (E), membrane (M), and nucleocapsid (N) proteins.^2,10,11^ Among them, S protein plays the most important roles in viral attachment, fusion and entry, and it serves as a target for development of antibodies, entry inhibitors and vaccines.^1,12–17^ The S protein mediates viral entry into host cells by first binding to a host receptor through the receptor-binding domain (RBD) in the S1 subunit and then fusing the viral and host membranes through the S2 subunit.^16,18,19^ SARS-CoV and MERS-CoV RBDs recognize different receptors. SARS-CoV recognizes angiotensin-converting enzyme 2 (ACE2) as its receptor, whereas MERS-CoV recognizes dipeptidyl peptidase 4 (DPP4) as its receptor.^20,21^ Similar to SARS-CoV, SARS-CoV-2 also recognizes ACE2 as its host receptor binding to viral S protein.^22^ Therefore, it is critical to define the RBD in SARS-CoV-2 S protein as the most likely target for the development of virus attachment inhibitors, neutralizing antibodies, and vaccines.

In this study, we identified the RBD fragment in SARS-CoV-2 S protein and found that the recombinant RBD protein bound strongly to human ACE2 (hACE2) and bat ACE2 (bACE2) receptors. In addition, it blocked the entry of SARS-CoV-2 and SARS-CoV into their respective hACE2-expressing cells, suggesting that it may serve as a viral attachment inhibitor against SARS-CoV-2 and SARS-CoV infection. Moreover, we demonstrated that SARS-CoV RBD-specific polyclonal antibodies cross-reacted with SARS-CoV-2 RBD protein and inhibited SARS-CoV-2 entry into hACE2-expressing cells. We have also shown that SARS-CoV RBD-specific polyclonal antibodies could cross-neutralize SARS-CoV-2 pseudovirus infection, suggesting the potential to develop SARS-CoV RBD-based vaccine for prevention of infection by SARS-CoV-2 and SARS-CoV.

## Results and discussion

**Fig. 1:**
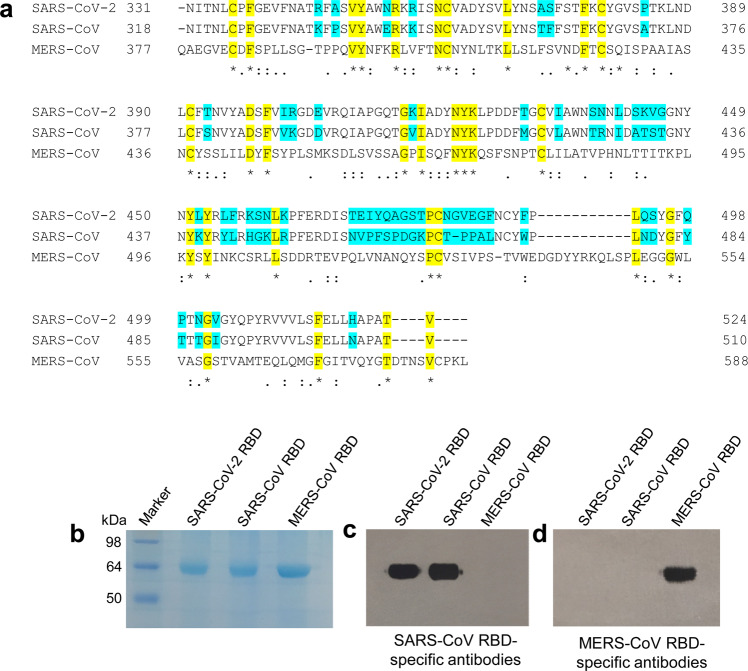
Characterization of SARS-CoV-2 RBD. **a** Multiple sequence alignment of RBDs of SARS-CoV-2, SARS-CoV, and MERS-CoV spike (S) proteins. GenBank accession numbers are QHR63250.1 (SARS-CoV-2 S), AY278488.2 (SARS-CoV S), and AFS88936.1 (MERS-CoV S). Variable amino acid residues between SARS-CoV-2 and SARS-CoV are highlighted in cyan, and conserved residues among SARS-CoV-2, SARS-CoV, and MERS-CoV are highlighted in yellow. Asterisks represent fully conserved residues, colons represent highly conserved residues, and periods represent lowly conserved residues. The alignment was performed using Clustal Omega. SDS-PAGE (**b**) and Western blot (**c**, **d**) analysis of SARS-CoV-2 RBD. The protein molecular weight marker (kDa) is indicated on the left. SARS-CoV and MERS-CoV RBDs were included as controls. Antisera (1:3,000 dilution) from mice immunized with SARS-CoV RBD (**c**) and MERS-CoV RBD (**d**) were used for Western blot analysis

By alignment of the RBD sequences of SARS-CoV and SARS-CoV-2, we identified the region of SARS-CoV-2 RBD at residues 331 to 524 of S protein (Fig. 1a). We then constructed a recombinant RBD protein containing codon-optimized RBD sequences with a C-terminal Fc of human IgG1 (hFc) using pFUSE-hIgG1-Fc2 expression vector, expressed the protein in mammalian cell 293T, and purified it from cell culture supernatant using protein A affinity chromatography. Similar to SARS-CoV and MERS-CoV RBD protein controls, SARS-CoV-2 RBD protein had high expression with strong purity (Fig. 1b). Notably, only SARS-CoV-2 and SARS-CoV RBDs were recognized by SARS-CoV RBD-specific, but not MERS-CoV RBD-specific, polyclonal antibodies (Fig. 1c), whereas only MERS-CoV RBD was recognized by MERS-CoV RBD-immunized polyclonal antibodies (Fig. 1d), suggesting the cross-reactivity of SARS-CoV RBD-specific antibodies with SARS-CoV-2 RBD protein.

**Fig. 2:**
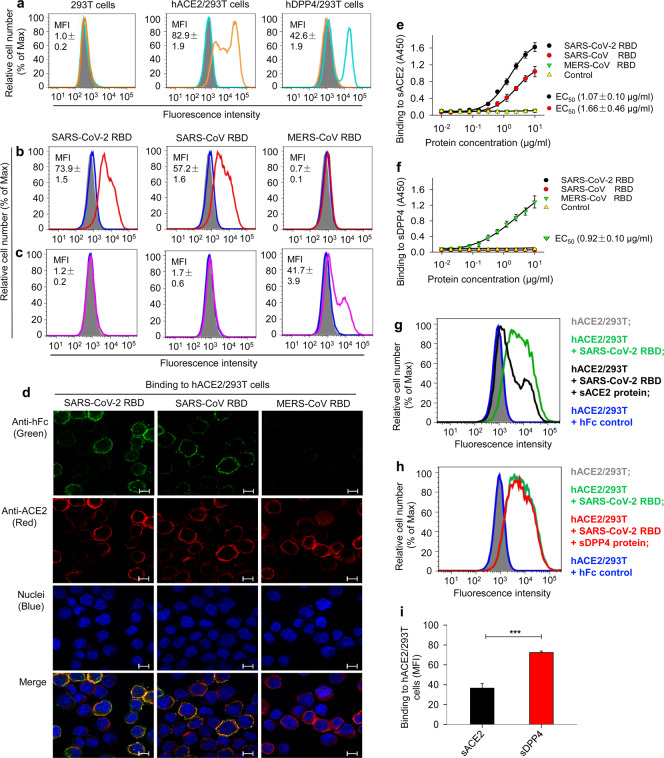
Detection of SARS-CoV-2 RBD binding to human ACE2 receptor. **a** Flow cytometry analysis of receptor expression in stable cell lines. (left panel) 293T cells alone expressed neither human ACE2 (hACE2) receptor (orange line) nor hDPP4 receptor (cyan line); (middle panel) hACE2-expressing 293T (hACE2/293T) cells expressed only hACE2 (orange line), but not hDPP4 (cyan line); (right panel) hDPP4-expressing 293T (hDPP4/293T) cells expressed only hDPP4 (cyan line), but not hACE2 (orange line). Mock-incubated cells (gray shading) were used as control. Representative images and median fluorescence intensity (MFI) ± standard error (s.e.m.) were shown (*n* = 4). **b**, **c** Flow cytometry analysis of SARS-CoV-2 RBD binding to cell-associated hACE2 receptor in hACE2/293T stable cell lines. SARS-CoV-2 RBD protein bound strongly to hACE2/293T cells (**b** (left panel, red line)), but not to hDPP4/293T cells (**c** (left panel, violet line)). SARS-CoV RBD protein bound to hACE2/293T cells (**b** (middle panel, red line)), but not to hDPP4/293T cells (**c** (middle panel, violet line)). MERS-CoV RBD protein did not bind to hACE2/293T cells (**b** (right panel, red line)), but rather bound to hDPP4/293T cells (**c** (right panel, violet line)). Human IgG Fc (hIgG-Fc, hereinafter hFc) protein-incubated cells (blue line) and mock-incubated cells (gray shading) were included as controls (**b**, **c**). Representative images and MFI ± s.e.m. were shown (*n* = 4). **d** Immunofluorescence detection of SARS-CoV-2 RBD binding to cell-associated hACE2 receptor in hACE2/293T cells. SARS-CoV-2 RBD protein (green) and SARS-CoV RBD protein (green), each of which was fused with a C-terminal hFc, were stained with FITC-labeled goat anti-human IgG antibody (1:500). hACE2 was stained with a goat-anti-hACE2 antibody (5 μg/ml) and Alexa-Fluor 647-labeled anti-goat antibody (red) (1:200). Fc-fused MERS-CoV RBD protein did not bind to hACE2, so only hACE2 (red), but not RBD (green), was detected in hACE2/293T cells. Nuclei were stained with 4’,6-diamidino-2-phenylindole (DAPI, blue). Scale bar: 10 μm. Representative images are shown. **e** Detection of dose-dependent binding of SARS-CoV-2 RBD protein to soluble hACE2 (sACE2) receptor by ELISA. The SARS-CoV-2 RBD binding to soluble hDPP4 (sDPP4) receptor (**f**), and the binding of both SARS-CoV RBD and MERS-CoV RBD proteins to sACE2 (**e**), or sDPP4 (**f**), were tested. Control: hFc protein. Data are presented as mean A450 ± s.e.m. (*n* = 4). 50% effective dose (EC_50_) was calculated for the binding between SARS-CoV-2 RBD (black) or SARS-CoV RBD (red) and hACE2 protein (**e**, sACE2), or the binding between MERS-CoV RBD and hDPP4 protein (sDPP4, green) (**f**). **g**–**i** Flow cytometry analysis of inhibition of SARS-CoV-2 RBD protein binding to hACE2/293T cells by sACE2. Binding of SARS-CoV-2 RBD to hACE2/293T cells (**g**, **h**, green line) was blocked by sACE2 (**g**, black line), but not by sDPP4 (**h**, red line). hFc protein-incubated cells (blue line) and mock-incubated cells (gray shading) were included as controls (**g**, **h**). Representative images are shown. **i** The blocking ability of sACE2 or sDPP4, as described above, was expressed as MFI ± s.e.m. (*n* = 4). Low MFI correlates with high blockage. Experiments were repeated twice and yielded similar results

Four experiments were performed to detect the binding between SARS-CoV-2 RBD and hACE2 receptor. First, we tested if stably transfected hACE2/293T cells expressed hACE2 by flow cytometry analysis. Since 293T cells alone did not express either hACE2 or hDPP4, they could not be recognized by anti-hACE2 or anti-hDPP4 antibodies (Fig. 2a (left panel)). Only hACE2/293T cells, but not hDPP4/293T cells, expressed hACE2, which was recognized by an anti-hACE2 antibody (Fig. 2a (middle panel)), whereas only hDPP4/293T cells, but not hACE2/293T cells, expressed hDPP4 and was, correspondingly, recognized by an anti-hDPP4 antibody (Fig. 2a (right panel)). These data confirmed the expression of hACE2 in hACE2/293T cells and the expression of hDPP4 in hDPP4/293T cells. Second, we used these hACE2/293T cells to detect the binding of SARS-CoV-2 RBD protein to cell-associated hACE2 by flow cytometry analysis and immunofluorescence staining. Similar to SARS-CoV RBD, SARS-CoV-2 RBD bound to hACE2/293T cells expressing hACE2 (Fig. 2b (left and middle panels)), but not to hDPP4/293T cells expressing hDPP4 (Fig. 2c (left and middle panels)). Furthermore, the binding between SARS-CoV-2 RBD and hACE2-expressing 293T cells was much stronger than the binding between SARS-CoV RBD and hACE2-expressing 293T cells (Fig. 2b (left and middle panels)). MERS-CoV RBD did not bind to hACE2-expressing 293T cells (Fig. 2b (right panel)), but rather bound to hDPP4-expressing 293T cells (Fig. 2c (right panel)). The results from immunofluorescence staining revealed positive signals for both hACE2 and hFc on hACE2/293T cells treated with SARS-CoV-2 RBD and SARS-CoV RBD, both of which contained a C-terminal hFc tag, whereas hACE2/293T cells treated with MERS-CoV RBD (containing a C-terminal hFc tag) showed positive signals for hACE2, but not for hFc, indicating that there is no binding of MERS-CoV RBD to the hACE2-expressing cells (Fig. 2d). These data suggest that SARS-CoV-2 RBD and SARS-CoV RBD can bind to cell-associated hACE2, but not to hDPP4. Third, we detected the binding of SARS-CoV-2 RBD to soluble hACE2 protein (sACE2) by ELISA. The results indicated that SARS-CoV-2 RBD bound to sACE2 in a dose-dependent manner and that the binding between SARS-CoV-2 RBD and sACE2 with 50% effective dose (EC_50_) of 1.07 μg/ml was stronger than that between SARS-CoV RBD and sACE2 (EC_50_: 1.66 μg/ml). In contrast, MERS-CoV RBD did not bind to sACE2 (Fig. 2e). While neither SARS-CoV-2 RBD nor SARS-CoV RBD bound to sDPP4, MERS-CoV RBD strongly bound to sDPP4 (EC_50_: 0.92 μg/ml) (Fig. 2f). These data suggest that both SARS-CoV-2 RBD and SARS-CoV RBD could bind to hACE2 in solution, but not to hDPP4 in solution. Fourth, flow cytometry analysis further indicated that the binding between SARS-CoV-2 RBD and cell-associated hACE2 receptor could be significantly blocked by sACE2 protein (Fig. 2g, i), but not by sDPP4 protein (Fig. 2h, i). Taken together, the above results confirm that the identified SARS-CoV-2 RBD could bind to both cell-associated and soluble hACE2 proteins.

**Fig. 3:**
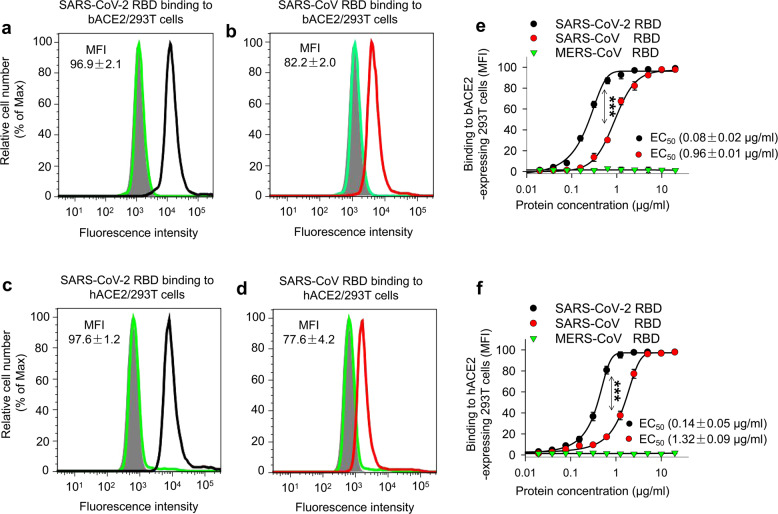
Comparison of SARS-CoV-2 RBD protein binding to human and bat ACE2 receptors. Flow cytometry analysis of SARS-CoV-2 RBD binding to hACE2 and bat ACE2 (bACE2) receptors in 293T cells transiently expressing hACE2 or bACE2. 293T cells were transiently transfected with hACE2 or bACE2 plasmid and incubated with SARS-CoV-2 RBD protein at various concentrations for analysis. SARS-CoV RBD and MERS-CoV RBD proteins were used as controls. Representative images of SARS-CoV-2 RBD protein (2.5 μg/ml) binding to bACE2/293T (**a**, black line), or hACE2/293T (**c**, black line), cells were shown. Binding of SARS-CoV RBD protein (2.5 μg/ml) to bACE2/293T (**b**, red line), or hACE2/293T (**d**, red line), cells were used as a comparison. MERS-CoV RBD protein (green line) and mock-incubated (gray shading) cells (**a**–**d**) were included as controls. **e**, **f** Dose-dependent binding of SARS-CoV-2 RBD protein to bACE2/293T (**e**), or hACE2/293T (**f**), cells by flow cytometry analysis. Significant differences between binding of SARS-CoV-2 RBD (black) and SARS-CoV RBD (red) to cell-associated bACE2 receptor (**e**), or hACE2 receptor (**f**) were identified based on the EC_50_ values. The data are presented as mean ± s.e.m. (*n* = 4). Experiments were repeated twice and yielded similar results

Like SARS-CoV and MERS-CoV, SARS-CoV-2 also originates from bats.^22–24^ Next, we detected the binding affinity of the identified SARS-CoV-2 RBD to bat ACE2 (bACE2) and compared this binding with that of SARS-CoV RBD. We transiently transfected a bACE2-expressing plasmid into 293T cells and included a hACE2-expressing plasmid as a control, followed by detection of fluorescence intensity 48 h later. Results indicated that SARS-CoV-2 RBD bound strongly to 293T-expressed bACE2 with intensity similar to that of its binding to 293T-expressed hACE2 (Fig. 3a, c), and that this binding occurred in a dose-dependent manner (Fig. 3e, f). In addition, the binding affinity between SARS-CoV-2 RBD and 293T-expressed bACE2 (EC_50_: 0.08 μg/ml) or hACE2 (EC_50_: 0.14 μg/ml) was significantly higher than that between SARS-CoV RBD and 293T-expressed bACE2 (EC_50_: 0.96 μg/ml) or hACE2 (EC_50_: 1.32 μg/ml) (Fig. 3b, d–f). Nevertheless, MERS-CoV RBD bound neither bACE2- nor hACE2-expressing 293T cells (Fig. 3). These data suggest that SARS-CoV-2 RBD can bind to both bACE2 and hACE2 with significantly stronger binding than that of SARS-CoV RBD to either bACE2 or hACE2, supporting the bat origin of SARS-CoV-2. These results may partially explain why SARS-CoV-2 is more transmissible than SARS-CoV.

**Fig. 4:**
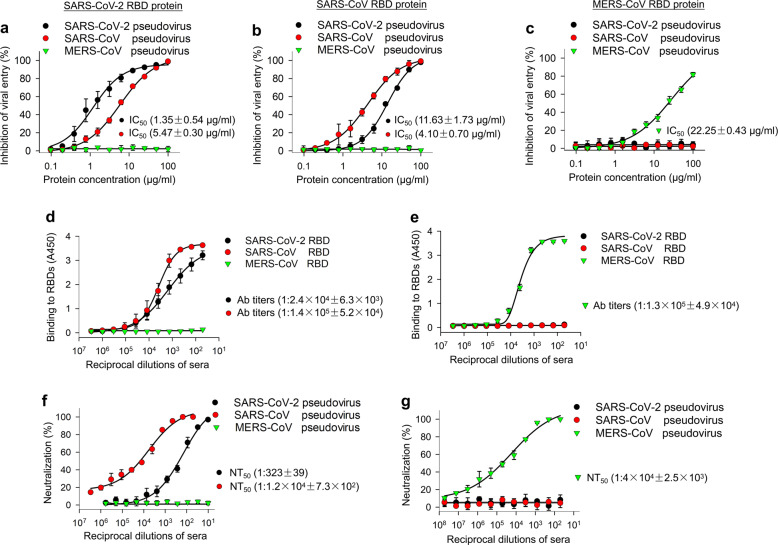
Ability of SARS-CoV-2 RBD to inhibit viral entry, as well as its cross-reactivity and cross-neutralizing activity with SARS-CoV. **a** Dose-dependent inhibition of SARS-CoV-2 RBD protein against pseudotyped SARS-CoV-2 entry into hACE2/293T cells. SARS-CoV and MERS-CoV RBDs, as well as hDPP4/293T cells, were included as controls. SARS-CoV-2 RBD protein inhibited entry of SARS-CoV-2 and SARS-CoV pseudoviruses into their respective target (hACE2/293T) cells (**a**), but not the entry of MERS-CoV pseudovirus into its target (hDPP4/293T) cells (**a**). SARS-CoV RBD protein inhibited both SARS-CoV-2 and SARS-CoV pseudovirus entry, but not MERS-CoV pseudovirus entry (**b**). MERS-CoV RBD inhibited neither SARS-CoV-2 nor SARS-CoV pseudovirus entry, but it did inhibit MERS-CoV pseudovirus entry (**c**). The data are presented as mean inhibition (%) ± s.e.m. (*n* = 4), and 50% inhibition concentration (IC_50_) was calculated for SARS-CoV-2 RBD (**a**, **b**, black), or SARS-CoV RBD (**a**, **b**, red), protein against SARS-CoV-2 pseudovirus and SARS-CoV pseudovirus and for MERS-CoV RBD protein (green) against MERS-CoV pseudovirus (**c**). **d** Cross-reactivity of SARS-CoV-2 RBD protein with SARS-CoV RBD-specific mouse sera by ELISA. Sera of mice immunized with mammalian cell-expressed SARS-CoV RBD protein^30^ were tested. Sera of mice immunized with mammalian cell-expressed MERS-CoV RBD protein^31^ were used as control. The data are presented as mean A450 ± s.e.m. (*n* = 4). The IgG antibody (Ab) titers were calculated as the endpoint dilution that remains positively detectable for SARS-CoV-2 RBD (black), or SARS-CoV RBD (red), binding to anti-SARS-CoV RBD sera (**d**) and for MERS-CoV RBD (green) binding to anti-MERS-CoV RBD sera (**e**). **f** Cross-neutralization of SARS-CoV RBD-immunized mouse sera against SARS-CoV-2 infection by pseudovirus neutralization assay. MERS-CoV RBD-immunized mouse sera were used as control. The data are presented as mean neutralization (%) ± s.e.m. (*n* = 4). 50% neutralizing antibody titers (NT_50_) were calculated against SARS-CoV-2 pseudovirus (black), or SARS-CoV pseudovirus (red), (**f**) infection in hACE2/293T target cells, as well as against MERS-CoV pseudovirus (green) (**g**) infection in hDPP4/293T cells. Experiments were repeated twice and yielded similar results

We then evaluated the potential of the identified SARS-CoV-2 RBD protein as an inhibitor of viral entry. To accomplish this, we first generated a pseudotyped SARS-CoV-2 by cotransfection of a plasmid encoding Env-defective, luciferase-expressing HIV-1 (pNL4-3.luc.RE) and a plasmid expressing S protein of SARS-CoV-2 into 293T cells, followed by collection of pseudovirus-containing supernatants. We then incubated serially diluted SARS-CoV-2 RBD protein with hACE2/293T target cells, followed by the addition of pseudovirus and detection of inhibitory activity of infection. With the capacity for only one-cycle infection, S protein-expressing pseudovirus cannot replicate in the target cells.^25,26^ Therefore, the inhibition of pseudovirus infection represents inhibition of viral entry, as mediated by viral S protein. As expected, SARS-CoV-2 RBD protein inhibited SARS-CoV-2 pseudovirus entry into hACE2-expressing 293T cells in a dose-dependent manner with 50% inhibition concentration (IC_50_) as low as 1.35 µg/ml. Interestingly, it also blocked the entry of SARS-CoV pseudovirus into hACE2-expressing 293T cells with IC_50_ of 5.47 µg/ml (Fig. 4a). Similarly, SARS-CoV RBD protein blocked the entry of both SARS-CoV pseudovirus and SARS-CoV-2 pseudovirus into hACE2-expressing 293T cells with IC_50_ of 4.1 and 11.63 µg/ml, respectively (Fig. 4b). In addition, neither SARS-CoV-2 RBD nor SARS-CoV RBD blocked the entry of MERS-CoV pseudovirus into hDPP4-expressing 293T cells (Fig. 4c). MERS-CoV RBD did not block the entry of SARS-CoV-2 pseudovirus or SARS-CoV pseudovirus into hACE2-expressing 293T cells, but it did block the entry of MERS-CoV pseudovirus into hDPP4-expressing 293T cells (IC_50_: 22.25 µg/ml) (Fig. 4a–c). These results suggest that SARS-CoV-2 RBD protein could be developed as an effective therapeutic agent against SARS-CoV-2 and SARS-CoV infection.

Since SARS-CoV-2 is more phylogenetically related to SARS-CoV than MERS-CoV,^22^ we further detected the cross-reactivity of SARS-CoV RBD-specific antibodies with SARS-CoV-2 RBD and cross-neutralizing activity of SARS-CoV RBD-specific antibodies against pseudotyped SARS-CoV-2. First, we performed an ELISA to detect the cross-reactivity of SARS-CoV RBD-immunized mouse sera with SARS-CoV-2 RBD. The results showed that SARS-CoV-2 RBD reacted strongly with anti-SARS-CoV RBD IgG with antibody titer of 1:2.4 × 10^4^ (Fig. 4d), but it did not react with anti-MERS-CoV RBD IgG (Fig. 4e). As expected, SARS-CoV RBD reacted strongly with anti-SARS-CoV RBD IgG (antibody titer: 1:1.4 × 10^5^) (Fig. 4d), but not with anti-MERS-CoV RBD IgG (Fig. 4e). MERS-CoV RBD did not react with anti-SARS-CoV RBD IgG (Fig. 4d), but instead reacted with anti-MERS-CoV RBD IgG (antibody titer: 1:1.3 × 10^5^) (Fig. 4e). Second, we performed a pseudovirus neutralization assay to detect the cross-neutralizing activity of SARS-CoV RBD-immunized mouse sera against SARS-CoV-2 pseudovirus infection. Results revealed that SARS-CoV RBD-specific antisera could neutralize SARS-CoV-2 pseudovirus infection with a neutralizing antibody titer of 1:323, while these antisera could neutralize SARS-CoV pseudovirus infection with higher neutralizing antibody titer (1:1.2 × 10^4^) (Fig. 4f). MERS-CoV RBD-inducing mouse sera only neutralized MERS-CoV pseudovirus infection in hDPP4-expressing cells with a neutralizing antibody titer of 1:4 × 10^4^ (Fig. 4g), but failed to neutralize infection by either SARS-CoV-2 pseudovirus or SARS-CoV pseudovirus (Fig. 4f). These data suggest that SARS-CoV RBD-specific antibodies can cross-react with SARS-CoV-2 RBD and cross-neutralize SARS-CoV-2 pseudovirus infection.

In summary, we have characterized the SARS-CoV-2 RBD protein which exhibits strong binding to its cell-associated and soluble ACE2 receptors with human and bat origin. This RBD protein also demonstrated significantly higher binding affinity to ACE2 than SARS-CoV RBD. SARS-CoV-2 RBD protein could block S protein-mediated SARS-CoV-2 pseudovirus and SARS-CoV pseudovirus entry into their respective ACE2 receptor-expressing target cells, suggesting the potential of SARS-CoV-2 RBD protein as a viral attachment or entry inhibitor against SARS-CoV-2 and SARS-CoV. SARS-CoV RBD-induced antibodies could cross-react with SARS-CoV-2 RBD and cross-neutralize SARS-CoV-2 pseudovirus infection, indicating that SARS-CoV RBD-specific antibodies may be used for treatment of SARS-CoV-2 infection and that either SARS-CoV RBD protein or SARS-CoV-2 RBD protein may be used as a candidate vaccine to induce cross-reactive or cross-neutralizing antibodies for prevention of SARS-CoV-2 or SARS-CoV infection. Taken together, this study provides an essential foundation for the design and development of SARS-CoV-2 RBD-based vaccines and therapeutics.

## Materials and methods

### Construction, expression, and purification of recombinant protein

The construction, expression, and purification of recombinant RBD proteins of SARS-CoV-2, SARS-CoV, and MERS-CoV were performed as previously described with some modifications.^27,28^ Briefly, genes encoding residues 331-524 of SARS-CoV-2 S protein, residues 318-510 of SARS-CoV S protein, or residues 377-588 of MERS-CoV S proteins, were amplified by PCR using codon-optimized SARS-CoV-2 S protein (GenBank accession number: QHR63250.1), SARS-CoV S protein (GenBank accession number: AY278488.2), or MERS-CoV S protein (GenBank accession number: AFS88936.1), as respective template, and fused into pFUSE-hIgG1-Fc2 expression vector (hereinafter named hFc; InvivoGen, San Diego, CA). The RBD proteins were expressed in human embryonic kidney (HEK)293T cells, secreted into cell culture supernatants, and purified by protein A affinity chromatography (GE Healthcare, Marlborough, MA).

### SDS-PAGE and Western blot

The purified RBD proteins were analyzed by SDS-PAGE and Western blot as previously described.^27,29^ Briefly, proteins were separated by 10% Tris-glycine SDS-PAGE and stained with Coomassie brilliant blue or transferred to nitrocellulose membranes. The blots were blocked with 5% fat-free milk in PBS containing 0.5% Tween-20 (PBST) for 2 h at 37 °C and further incubated with SARS-CoV RBD-specific polyclonal antibody (mouse sera, 1:3,000),^30^ or MERS-CoV RBD-specific antibody (mouse sera, 1:3,000),^31^ overnight at 4 °C. The blots were then incubated with horseradish peroxidase (HRP)-conjugated goat anti-mouse IgG (1:5,000, Thermo Fisher Scientific, Waltham, MA) for 1 h at room temperature and then visualized with ECL Western blot substrate reagents and Amersham Hyperfilm (GE Healthcare).

### Flow cytometry analysis

Flow cytometry analysis was performed to detect the binding of SARS-CoV-2 RBD protein to hACE2 receptor in 293T cells stably expressing hACE2 (hACE2/293T).^26,32^ SARS-CoV and MERS-CoV RBDs, as well as 293T cells stably expressing hDPP4 receptor (hDPP4/293T), were used as controls. Briefly, cells were incubated with respective RBD of SARS-CoV-2, SARS-CoV, or MERS-CoV containing a C-terminal hFc at 20 μg/ml for 30 min at room temperature, which was followed by incubation with FITC-labeled goat anti-human IgG antibody (1:500; Thermo Fisher Scientific) for 30 min and analyzed by flow cytometry. The blockage of RBD-receptor binding was performed by incubation of soluble human ACE2 (sACE2; 5 μg/ml; R&D Systems, Minneapolis, MN) receptor with respective RBD of SARS-CoV-2, SARS-CoV, or MERS-CoV (20 μg/ml), followed by the same procedure as that described above. hIgG-Fc protein (hFc: 20 μg/ml), or soluble human DPP4 (sDPP4; 5 μg/ml; R&D Systems) receptor, was included as control.

Detection of hACE2 protein expression in hACE2/293T, or hDPP4 protein expression in hDPP4/293T, stable cell lines was performed by flow cytometry analysis, as described above, except that the cells were sequentially incubated with hACE2- or hDPP4-specific goat antibody (0.5 μg/ml; R&D Systems) at room temperature for 20 min and FITC-labeled anti-goat IgG antibody (1:200; Abcam, Cambridge, MA) for 1 h at 4 °C.

Flow cytometry analysis was also performed to detect the binding between SARS-CoV-2 RBD and hACE2, or bat-ACE2 (bACE2), receptor in transiently transfected 293T cells. Briefly, 293T cells were transfected with hACE2 or bACE2 plasmid using the calcium phosphate method, and 48 h later, they were incubated with SARS-CoV-2 RBD protein at various concentrations for 30 min at room temperature. SARS-CoV and MERS-CoV RBDs were included as controls. After staining with FITC-conjugated goat anti-human IgG antibody (1:500; Thermo Fisher Scientific), the mixture was analyzed by flow cytometry as described above.

### Immunofluorescence staining

This was performed to detect the binding between SARS-CoV-2 RBD and hACE2 receptor in hACE2/293T stable cell lines.^33^ SARS-CoV and MERS-CoV RBDs were used as controls. Briefly, cells were sequentially incubated with Fc-fused SARS-CoV-2, SARS-CoV, or MERS-CoV RBD (20 μg/ml) and hACE2-specific goat antibody (5 μg/ml) for 30 min at room temperature. After three washes, the cells were incubated with FITC-labeled goat anti-human IgG (Fc) antibody (1:500; Thermo Fisher Scientific), or Alexa-Fluor 647-labeled anti-goat antibody (1:200 dilution; Abcam) for 30 min at room temperature. The nuclei were stained with 4’,6-diamidino-2-phenylindole (DAPI) for 5 min and mounted in VectaMount Permanent Mounting Medium. The samples were imaged on a confocal microscope (Zeiss LSM 880), and the images were prepared using the ZEN software.

### ELISA

ELISA was performed to detect the binding of SARS-CoV-2 RBD protein to sACE2 receptor, as previously described.^27,32,34^ SARS-CoV and MERS-CoV RBDs, as well as sDPP4 protein, were used as controls. Briefly, ELISA plates were precoated with SARS-CoV-2, SARS-CoV, or MERS-CoV RBD (1 μg/ml) overnight at 4 °C and blocked with 2% fat-free milk in PBST for 2 h at 37 °C. Serially diluted sACE2, or sDPP4, protein was added to the plates and incubated for 2 h at 37 °C. After four washes, the bound protein was detected using hACE2- or hDPP4-specific goat antibody (0.5 μg/ml, R&D system) for 2 h at 37 °C, followed by incubation with HRP-conjugated anti-goat IgG antibody (1:5,000, Thermo Fisher Scientific) for 1 h at 37 °C. The reaction was visualized by addition of substrate 3,3’,5,5’-Tetramethylbenzidine (TMB) (Sigma, St. Louis, MO) and stopped by H_2_SO_4_ (1 N). The absorbance at 450 nm (A450) was measured by an ELISA plate reader (Tecan, San Jose, CA).

The cross-reactivity of SARS-CoV-2 RBD protein to SARS-CoV RBD-specific antibody was performed by coating ELISA plates with SARS-CoV-2 RBD (1 μg/ml), as well as SARS-CoV RBD or MERS-CoV RBD (as controls, 1 μg/ml), followed by sequential incubation with serially diluted SARS-CoV RBD- or MERS-CoV RBD-immunized mouse sera and HRP-conjugated anti-mouse IgG (1:5,000; Thermo Fisher Scientific) antibodies.

### Pseudovirus neutralization and inhibition assays

SARS-CoV-2 pseudovirus was generated, as previously described, with some modifications.^25,27,29^ Briefly, 293T cells were cotransfected with a plasmid encoding Env-defective, luciferase-expressing HIV-1 genome (pNL4-3.luc.RE) and a plasmid encoding SARS-CoV-2 S protein using the calcium phosphate method. SARS-CoV and MERS-CoV pseudoviruses were packaged as controls. The transfected medium was changed into fresh Dulbecco’s Modified Eagle’s Medium (DMEM) 8 h later, and pseudovirus-containing supernatants were collected 72 h later for single-cycle infection in target cells. Pseudovirus neutralization assay was then performed by incubation of SARS-CoV-2, SARS-CoV, or MERS-CoV pseudovirus with serially diluted SARS-CoV RBD- or MERS-CoV RBD-immunized mouse sera for 1 h at 37 °C, followed by addition of the mixture into hACE2/293T (for SARS-CoV-2 pseudovirus and SARS-CoV pseudovirus) or hDPP4/293T (for MERS-CoV pseudovirus) target cells. Fresh medium was added 24 h later, and the cells were lysed 72 h later in cell lysis buffer (Promega, Madison, WI). The lysed cell supernatants were incubated with luciferase substrate (Promega) and detected for relative luciferase activity using the Infinite 200 PRO Luminator (Tecan). The 50% MERS pseudovirus neutralizing antibody titer (NT_50_) was calculated using the CalcuSyn computer program, as previously described.^29,35^

Inhibition of pseudovirus entry by SARS-CoV-2 RBD protein was carried out, as previously described, with some modifications.^31^ Briefly, SARS-CoV-2 RBD protein at serial dilutions was incubated with hACE2/293T target cells for 1 h at 37 °C. After removing medium containing the protein, the cells were infected with SARS-CoV-2 pseudovirus. SARS-CoV RBD and MERS-CoV RBD, as well as SARS-CoV pseudovirus and MERS-CoV pseudovirus, were used as controls. Fresh medium was added 24 h later, and the cells were lysed and analyzed, as described above. The 50% inhibitory concentration (IC_50_) of the RBD protein was calculated using the CalcuSyn computer program, as described above.

### Statistical analysis

Values were expressed as mean and standard error (s.e.m). Statistical significance between different groups was calculated by GraphPad Prism Statistical Software. Two-tailed Student’s *t* test was used. ^∗∗∗^ represents *P* < 0.001.

## References

[1] Du L, He Y, Zhou Y, Liu S, Zheng BJ, Jiang S (2009). The spike protein of SARS-CoV-a target for vaccine and therapeutic development. Nat. Rev. Microbiol..

[2] Wang N, Shang J, Jiang S, Du L (2020). Subunit vaccines against emerging pathogenic human coronaviruses. Front. Microbiol..

[3] World Health Organization. Naming the coronavirus disease (COVID-19) and the virus that causes it. https://www.who.int/emergencies/diseases/novel-coronavirus-2019/technical-guidance/naming-the-coronavirus-disease-(covid-2019)-and-the-virus-that-causes-it (2020).

[4] Zhong NS (2003). Epidemiology and cause of severe acute respiratory syndrome (SARS) in Guangdong, People’s Republic of China, in February, 2003. Lancet.

[5] Zaki AM, van Boheemen S, Bestebroer TM, Osterhaus AD, Fouchier RA (2012). Isolation of a novel coronavirus from a man with pneumonia in Saudi Arabia. N. Engl. J. Med..

[6] Zhu N (2020). A novel coronavirus from patients with pneumonia in China, 2019. N. Engl. J. Med..

[7] World Health Organization. Coronavirus disease 2019 (COVID-19)-Situation report-45. https://www.who.int/docs/default-source/coronaviruse/situation-reports/20200305-sitrep-45-covid-19.pdf?sfvrsn=ed2ba78b_2 (2020).

[8] Gorbalenya, A. E., et al. The species Severe acute respiratory syndrome-related coronavirus: classifying 2019-nCoV and naming it SARS-CoV-2. *Nat. Microbiol.*10.1038/s41564-020-0695-z (2020). 10.1038/s41564-020-0695-z PMC7095448 32123347

[9] Jiang Shibo, Shi Zhengli, Shu Yuelong, Song Jingdong, Gao George F, Tan Wenjie, Guo Deyin (2020). A distinct name is needed for the new coronavirus. The Lancet.

[10] Du L, Tai W, Zhou Y, Jiang S (2016). Vaccines for the prevention against the threat of MERS-CoV. Expert Rev. Vaccines.

[11] Zhou Y, Jiang S, Du L (2018). Prospects for a MERS-CoV spike vaccine. Expert Rev. Vaccines.

[12] Du L, Yang Y, Zhou Y, Lu L, Li F, Jiang S (2017). MERS-CoV spike protein: a key target for antivirals. Expert Opin. Ther. Targets.

[13] Lu L (2014). Structure-based discovery of Middle East respiratory syndrome coronavirus fusion inhibitor. Nat. Commun..

[14] Du L (2016). Introduction of neutralizing immunogenicity index to the rational design of MERS coronavirus subunit vaccines. Nat. Commun..

[15] He Y, Li J, Heck S, Lustigman S, Jiang S (2006). Antigenic and immunogenic characterization of recombinant baculovirus-expressed severe acute respiratory syndrome coronavirus spike protein: implication for vaccine design. J. Virol..

[16] Liu S (2004). Interaction between heptad repeat 1 and 2 regions in spike protein of SARS-associated coronavirus: implications for virus fusogenic mechanism and identification of fusion inhibitors. Lancet.

[17] Wang Q, Wong G, Lu G, Yan J, Gao GF (2016). MERS-CoV spike protein: targets for vaccines and therapeutics. Antivir. Res..

[18] Li F, Li W, Farzan M, Harrison SC (2005). Structure of SARS coronavirus spike receptor-binding domain complexed with receptor. Science.

[19] Lu G (2013). Molecular basis of binding between novel human coronavirus MERS-CoV and its receptor CD26. Nature.

[20] Li W (2003). Angiotensin-converting enzyme 2 is a functional receptor for the SARS coronavirus. Nature.

[21] Raj VS (2013). Dipeptidyl peptidase 4 is a functional receptor for the emerging human coronavirus-EMC. Nature.

[22] Zhou, P., et al. A pneumonia outbreak associated with a new coronavirus of probable bat origin. *Nature* **579**, 270–273 (2020). 10.1038/s41586-020-2012-7 PMC7095418 32015507

[23] Li W (2005). Bats are natural reservoirs of SARS-like coronaviruses. Science.

[24] Yang Y (2014). Receptor usage and cell entry of bat coronavirus HKU4 provide insight into bat-to-human transmission of MERS coronavirus. Proc. Natl Acad. Sci. U. S. A..

[25] Zhao G (2013). A safe and convenient pseudovirus-based inhibition assay to detect neutralizing antibodies and screen for viral entry inhibitors against the novel human coronavirus MERS-CoV. Virol. J..

[26] Du L (2014). A conformation-dependent neutralizing monoclonal antibody specifically targeting receptor-binding domain in Middle East respiratory syndrome coronavirus spike protein. J. Virol..

[27] Tai W (2017). Recombinant receptor-binding domains of multiple Middle East respiratory syndrome coronaviruses (MERS-CoVs) induce cross-neutralizing antibodies against divergent human and camel MERS-CoVs and antibody escape mutants. J. Virol..

[28] Ma C (2014). Searching for an ideal vaccine candidate among different MERS coronavirus receptor-binding fragments-the importance of immunofocusing in subunit vaccine design. Vaccine.

[29] Du L (2009). Recombinant receptor-binding domain of SARS-CoV spike protein expressed in mammalian, insect and E. coli cells elicits potent neutralizing antibody and protective immunity. Virology.

[30] Du L (2010). A 219-mer CHO-expressing receptor-binding domain of SARS-CoV S protein induces potent immune responses and protective immunity. Viral Immunol..

[31] Tai W (2016). A recombinant receptor-binding domain of MERS-CoV in trimeric form protects human dipeptidyl peptidase 4 (hDPP4) transgenic mice from MERS-CoV infection. Virology.

[32] Du L (2013). Identification of a receptor-binding domain in the S protein of the novel human coronavirus Middle East respiratory syndrome coronavirus as an essential target for vaccine development. J. Virol..

[33] Tai W (2019). Transfusion-transmitted Zika virus infection in pregnant mice leads to broad tissue tropism with severe placental damage and fetal demise. Front. Microbiol..

[34] Chen WH (2014). Yeast-expressed recombinant protein of the receptor-binding domain in SARS-CoV spike protein with deglycosylated forms as a SARS vaccine candidate. Hum. Vaccin. Immunother..

[35] Chou TC (2006). Theoretical basis, experimental design, and computerized simulation of synergism and antagonism in drug combination studies. Pharmacol. Rev..

